# Case report: Increased efficacy of cetuximab after pembrolizumab failure in cutaneous squamous cell carcinoma

**DOI:** 10.3389/fonc.2024.1385094

**Published:** 2024-05-08

**Authors:** Renee Morecroft, Jordan Phillipps, Alice Zhou, Omar Butt, Karam Khaddour, Tanner Johanns, George Ansstas

**Affiliations:** ^1^ Division of Medical Oncology, Department of Medicine, Washington University in St. Louis, St. Louis, MO, United States; ^2^ Department of Medical Oncology, Dana-Farber Cancer Institute, Harvard Medical School, Boston, MA, United States

**Keywords:** cutaneous squamous cell carcinoma, cutaneous oncology, cetuximab, antiepidermal growth factor receptor, pembrolizumab, immunotherapy, case report

## Abstract

Immunotherapy is the first-line option for treating advanced cutaneous squamous cell carcinoma (cSCC). However, up to half of patients experience no benefit and treatment resistance, warranting newer therapeutic approaches. Combinatory approaches, including cetuximab, may help overcome immunotherapy resistance and improve response rates in advanced cSCC. We report three cases of metastatic cSCC that achieved significant clinical responses after cetuximab therapy following initial progression on pembrolizumab. We have retrospectively reviewed these cases at a single academic center between 2018 and 2023. All patients initially progressed on pembrolizumab, after which cetuximab (mono- or combination therapy) was added with two complete responses and one partial response. Initial responses were noted within 2 to 7 months of starting cetuximab. While the benefit of cetuximab and immunotherapy in head-and-neck squamous cell carcinoma has growing evidence, information regarding cSCC remains limited. This study adds three cases to the underreported literature on treating advanced cSCC with cetuximab after initially failing immunotherapy.

## 1 Introduction

Despite the excellent overall prognosis of cutaneous squamous cell carcinoma (cSCC), approximately 3-7% of patients will experience locoregional or distant metastasis which portends a poor prognosis (1). Unfortunately, cytotoxic chemotherapy and epidermal growth factor receptor (EGFR) therapies, such as cetuximab, have had limited success due to a short durable response. First-line therapy has therefore shifted to immune checkpoint inhibitors (ICI), such as programmed cell death protein 1 (PD-1) inhibitors, demonstrating a mixed response with half of patients experiencing no benefit or progression (2, 3). Improving the ICI response rate may lie in cetuximab use post-ICI rather than pre-ICI due to greater efficacy (**Table 1**). Here, we report three cases of advanced cSCC initially unresponsive to anti-PD-1 therapy but improved significantly with subsequent cetuximab therapy and provide a comprehensive review of the available literature on this subject.

**Table 1:** Current literature assessing cetuximab use after anti-PD-1 therapy in cutaneous squamous cell carcinoma.

Author	Study Design	Number of Patients	Treatment Regimen	IO included (Y/N)	EGFR Inhibitor Dose	Response Pattern	RFS/PFS	OS	Reference
**Bossi P et al.**	Phase-2 single- arm clinical trial	21	cetuximab + pembrolizumab after pembrolizumab progression	Y	cetuximab 400 mg/m^2^ loading dose, then 250 mg/m^2^ day 1,8, 15 of a 3-week schedule	38% ORR	12 months	Not reached	(4)
**Marin- Acevedo JA et al., 2023**	Retrospective cohort study	23 (Cohort A & B= 13; Cohort C=10)	Cohort A&B = cetuximab after ICI progression Cohort C = cetuximab with no prior ICI	N	unknown	Cohort A & B= 53.9% ORR (CR= 7.7%; PR=46.2%)	17.3 months	Not reached	(1)
**Chen A et al.**	Case report	1	cetuximab+ nivolumab	Y	unknown	100% CR	12 months	Not reached	(5)
**Hsu E et al.**	Case Series	3	panitumumab added to anti-PD-1 antibody therapy after progression with 3 monthly interval scans	Y	Panitumumab 6mg/kg every 2 weeks	100% CR	Patient 1: 25 months Patient 2: 18+ months Patient 3: 14+ plus	Not stated	(6)
**Chang M et al.**	Case series	18	cetuximab induction and concurrent radiotherapy	N	cetuximab 400 mg/m² loading dose then 250 mg/m² weekly throughout radiation	83.2% ORR (55.5% CR; 27.7% PR)	Median PFS= 21.6 (61% at 1 year & 40% at 2 years	Median OS=60.7 % at 3.5 to 62.5 months	(7)
**Maubec E et al.**	Phase-2 single- arm clinical trial	36	Cetuximab	N	cetuximab 400 mg/m^2^ loading dose the 250 mg/m^2^ weekly doses	59% DCR	4.1 months	8.1 months	(8)

Immunotherapy (IO) Recurrence-Free Survival (RFS), Progression- Free Survival (PFS), Overall Survival (OS), Complete Response (CR), Partial Response (PR), Overall Response Rate (ORR), Local Control Rate (LCR), Disease Control Rate (DCR).

Y, yes; N, no.

## 2 Materials and methods

We identified three patients from 2018 to 2023 at the Siteman Cancer Center of Washington University School of Medicine who presented for the management of advanced metastatic cSCC. All patients initially progressed on pembrolizumab, after which cetuximab was added with significant clinical response (two complete responses [CR] and one partial response [PR]). All patients were amenable to their respective treatment plans. Treatments were well-tolerated overall. Patient demographics and clinical data are reported in **Table 2**.

**Table 2:** Patient demographics and Clinical Data.

	Patient 1	Patient 2	Patient 3
**Age**	67	61	36
**Gender**	Male	Male	Male
**Race**	White	White	Black
**cSCC Site**	Right lower abdomen	Left pre-auricle	Right back
**Initial therapy**	Pembrolizumab	Pembrolizumab	Pembrolizumab
**Initial Response after** **Pembrolizumab**	PD	PD	PD
**Time to PD after Pembrolizumab** **(months)**	2.6	2.0	6.8
**Subsequent Therapy**	Cetuximab	Cetuximab, Carboplatin	Cetuximab, Carboplatin
**Initial Response** **after Cetuximab**	PR	CR	PR
**Time to Initial** **response after Cetuximab (months)**	7.0	1.9	2.1
**Treatment at the** **time of Initial Response**	Pembrolizumab, Cetuximab	Cetuximab, Carboplatin	Pembrolizumab, Cetuximab, Carboplatin
**Best Response after** **Cetuximab**	PR	CR	CR
**Total Length of Therapy on** **Cetuximab (months)**	10.0	28.2	27.6
**Disease-Free** **Survival (months)**	N/A	26.3	10.7
**Follow-up Time** **(months)**	13.3	31.3	46.1
**Immune-related Adverse Event (IRAE)**	None	None	Colitis (grade 3; quickly resolved with steroids with successful rechallenge)
**Cetuximab Adverse Events**	Rash (grade 1; self- resolved)	None	Acneiform Rash (grade 1; treated with topical steroids)

cSCC, cutaneous squamous cell carcinoma; PD, progression of disease; PR, partial response; CR, complete response; IRAE, immune-related adverse event.

N/A, Not Applicable.

### 2.1 Patient 1

**Figure 1:**
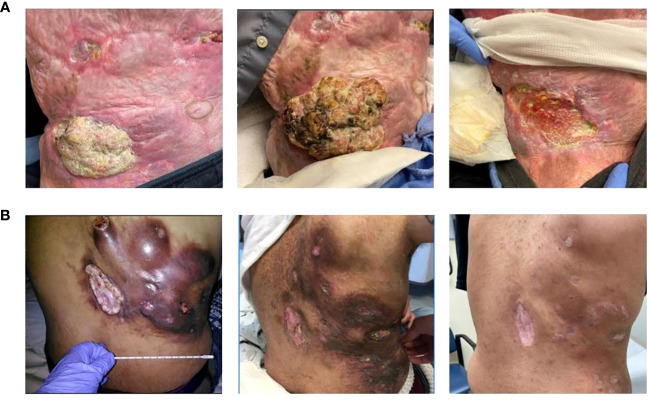
Cutaneous Squamous cell carcinoma Clinical Presentation. **(A)** Patient 1 (from left to right): Initial presentation at baseline; progression of disease after 3 months of pembrolizumab; partial response after 7 months of cetuximab. **(B)** Patient 3 (from left to right): Initial presentation at baseline; partial response after 2 months of cetuximab; complete response after 28 months of cetuximab.

A 67-year-old Caucasian male presented with right lower quadrant abdominal invasive, unresectable cSCC. He had a history of significant burns (>80% of total body surface area) during a fire approximately 40 years earlier, complicated by chronic wounds and Marjolijn ulcers requiring right arm amputation and surgical resection in the central abdominal area (Stage III – pT3, pN1, cM0). After three months of pembrolizumab, he exhibited marked disease progression (increased size of both the pre-existing abdominal lesion and several subcutaneous nodules posterior to the right iliac bone, in addition to new left axillary lymphadenopathy) resulting in the addition of Cetuximab (500 mg/m2 biweekly). Restaging scans at seven months demonstrated significant clinical and radiographic responses (80-90% reduction of tumor burden with near complete resolution of nodal metastases; **Figure 1A**. His clinical PR has persisted at 10 months since cetuximab initiation, and he continued pembrolizumab and cetuximab at the last follow-up. He had no significant adverse events aside from a self-resolving Grade 1 rash.

### 2.2 Patient 2

A 61-year-old Caucasian male presented for evaluation of recurrent metastatic cSCC. His medical history was notable for end-stage renal disease treated with a renal transplant five years earlier, which has since been complicated by multiple basal cell carcinomas (BCC) and cSCC in sun-exposed areas, requiring local therapies. He had an excision of a cSCC of the left pre-auricular region with parotid gland involvement three years prior and additional excision with adjuvant radiation therapy due to positive lymph nodes (stage IV - pT3, pN2a, cM0). Subsequent restaging scans revealed lung and mediastinal metastases. Pembrolizumab was initiated after considering the potential risk of allograft rejection. His tacrolimus dosage was decreased from 2 to 1 mg daily (while continuing low-dose 5 mg prednisone daily). Pembrolizumab was discontinued due to disease progression upon restaging scans two months later. He then started concurrent carboplatin (Target AUC = 5 mg/mL/min monthly; 6 cycles total) and cetuximab (250 mg/m2 weekly). Ultimately, a CR after two months was achieved, which has sustained for 26 months. Carboplatin was discontinued three months after his CR. He remains on cetuximab monotherapy at the last follow-up, ongoing for 28 months since treatment initiation. He has tolerated therapy well with no adverse events.

### 2.3 Patient 3

A 36-year-old African-American male presented with a large right lower-mid back wound initially noted as a small “mole” five years prior. Excisional biopsy revealed well-differentiated cSCC (stage IV – pT3, pN2c, cM0). Given his unresectable mass and nodal metastases, pembrolizumab was started. Restaging scans at seven months revealed disease progression, prompting the addition of carboplatin (Target AUC = 5 mg/mL/min monthly; 15 cycles total) and cetuximab (250 mg/m2 weekly) to pembrolizumab therapy. Two months later (i.e. nine after pembrolizumab initiation), PR was first observed. Pembrolizumab was then held for two months for mild, biopsy-proven checkpoint-induced colitis managed with a steroid taper. Restaging scans at 28 months after cetuximab initiation demonstrated a CR, prompting discontinuation of treatment (**Figure 1B**). He remains disease-free at the last follow-up 11 months from treatment discontinuation (39 months from original cetuximab initiation). He had no significant adverse events except for a grade 1 acneiform rash controlled with topical hydrocortisone and oral minocycline.

## 3 Diagnostic assessment

In this case series, three adults with advanced cSCC achieved clinical and radiographic responses (1 PR and 2 CR) with cetuximab (either as a monotherapy or combination therapy) after progression on pembrolizumab. Treatment was well-tolerated with rapid responses within seven months of initiating cetuximab.

## 4 Discussion

Cutaneous SCC is an immunogenic cancer capable of immune surveillance escape via an immune-tolerant microenvironment (9). Upregulation of inhibitory immune checkpoint expression, such as programmed death ligand 1 (PD-L1) promotes T-cell exhaustion. PD-1 inhibitors, such as pembrolizumab, promote T-cell activation via PD-1 blockade thus rejuvenating tumor-specific cytotoxic T cells in the tumor microenvironment (TME) and an anti-tumor effect (9). Cetuximab (EGFR inhibitor) also exerts anti-tumor activity through several immune-based mechanisms including natural killer (NK) cell-mediated killing via antibody-dependent cytotoxicity (ADCC) and tumor cell death via cytotoxic T-cell priming (10).

The KEYNOTE-629 and CARSKIN trials ushered in the pembrolizumab era for recurrent and advanced metastatic cSCC (2). However, almost half of patients lack benefit from PD-1 blockade thus warranting investigation into other therapies (9). A small phase 2 trial showed a 69% (n=25) disease control rate (DCR) after 6 weeks of cetuximab first-line monotherapy, with 8 patients (22%) achieving PR and 2 patients (6%) achieving CR (8). The median duration of response (DOR) was 6.8 months (95% CI, 4.1 to 8.3 months), sustained for 2.5 years after therapy cessation (8). Despite this, second-line cetuximab therapy may have a role in the setting of PD-1 blockade therapy failure (1). A single institution retrospective review (n=13) found that cetuximab use immediately after PD-1 progression had a greater and longer-lasting overall response compared to cetuximab use before PD-1 blockade from historical data (1). This study showed a 6-month DCR of 77% and an objective response rate (ORR) of 54% with 6 PR and 1 CR (1). In the I-Tackle phase 2 trial, patients with locally advanced or metastatic cSCC who progressed or had stable disease on pembrolizumab received cetuximab while continuing pembrolizumab therapy (n=23/43) (4). Cetuximab reversed the resistance to pembrolizumab (the response rate to combinatory treatment was nearly double that of cetuximab monotherapy), suggesting a rationale in adding cetuximab to pembrolizumab-refractory cSCC. However, while promising, the underlying mechanism is unclear (4). Of relevance, all patients had a molecular or clinical basis for their initial poor response to immunotherapy. Patient 1 had a low tumor mutational burden (TMB) of 7 mutations/Mb; moreover, immunotherapy response in patients with Marjolin ulcers is not well described (although presumably, these patients are less likely to have high TMB compared to traditionally sun-induced cSCC). Patient 3 also had a low TMB of 1.3 mutations/Mb, while Patient 2 is chronically immunosuppressed due to their history of renal transplantation. Nonetheless, our study further supports the use of cetuximab after PD-1 inhibitor failure either as a monotherapy or in combination with other systemic treatments (chemotherapy or immunotherapy).

Cetuximab and PD-1 inhibition synergism has been demonstrated in head and neck squamous cell carcinoma (HNSCC) with PD-1 blockade and cetuximab contributing to anti-tumor NK cell response (11). PD-1 inhibition also increased cetuximab-mediated NK cell activation and cytotoxicity, preferentially targeting HNCSS with high PD-L1 expression. These patients had an increased number of PD-1+ NK cells, correlating with diminished NK cell activity (11). PD-L1 expression correlated with a lack of response to cetuximab monotherapy, whereas concurrent administration with a PD-1 inhibitor (nivolumab) successfully reversed NK cell dysfunction and enhanced cetuximab-mediated ADCC *in vitro* (11). Thus, there may be synergy between PD-1 inhibitors and cetuximab in immune system priming to target cancer cells. PD-1 blockade of PD-1+ NK cells allows cetuximab to activate and restore their activity, thus enhancing their cytotoxicity. A group observed HNSCC patients treated with cetuximab after immunotherapy to have a longer median overall survival (OS) (11.3 months vs 8.0 months) and ORR (37.5% vs 19.0%, respectively), than those without post-immunotherapy cetuximab (10). Relatedly, separate treatment avenues exist whereby the effect of cetuximab may be potentiated following immunotherapy. A recent retrospective cohort study assessing combinatory cetuximab and radiation in locally advanced cSCC observed great outcomes (ORR 83.2% with 55.5% CR and 27.7% PR) in a small number of patients (n=18) (7). While promising, further prospective studies are required to investigate this relationship in patients who have received or are receiving immunotherapy. **Table 1** pools key information from cited studies assessing cetuximab use after PD-1 inhibition.

While the benefit of cetuximab and immunotherapy in HNSCC has growing evidence, information remains limited in cSCC. One report observed clinical remission of auricular cSCC with combinatory nivolumab and cetuximab (5). This benefit was also observed with other anti-EGFR inhibitors – several metastatic cSCC patients refractory to immunotherapy achieved durable CRs after the addition of panitumumab (6) Our report is unique as it adds three cases to this underreported body of evidence. Interestingly, it does not appear that cetuximab before PD-1 inhibition offers the same benefits. In the same study, patients with cetuximab use before PD-1 inhibition had significantly worse progression-free survival (PFS) (3.0 months vs 4.4 months; p=0.024 after multivariable analysis) and median OS (13.6 months vs 19.8 months; p=0.003 after multivariable analysis) than patients who did not have prior cetuximab use (10). Additionally, the ORR (to PD-1 inhibition) in this group was lower than that of patients who did not have prior cetuximab use (19.0% vs 27.2%, respectively; p=0.44), although this difference was not statistically significant (10). One hypothesis is that cetuximab dampens the response of subsequent immunotherapy by down-regulating PD-L1 expression and recruiting regulatory T cells (12). Taken together, immunotherapy alters the TME in a way that amplifies cetuximab’s effect, whereas the reverse may not be true.

In conclusion, this study supports a growing body of evidence on the efficacy of cetuximab (and other anti-EGFR agents) in treating advanced cSCC otherwise unresponsive to PD-1 inhibition. Our study is limited by a small sample size and its retrospective nature. The efficacy of cetuximab after progression on PD-1 blockade therapy in treating advanced cSCC requires investigation by larger, randomized controlled trials to further understand its mechanism of action and establish this approach as the standard of care.

## Data availability statement

The original contributions presented in the study are included in the article/supplementary material. Further inquiries can be directed to the corresponding author.

## Ethics statement

Ethical approval was not required for the study involving humans in accordance with the local legislation and institutional requirements. Written informed consent to participate in this study was not required from the participants or the participants’ legal guardians/next of kin in accordance with the national legislation and the institutional requirements. Written informed consent was obtained from the individual(s) for the publication of any potentially identifiable images or data included in this article.

## Author contributions

RM: Investigation, Project administration, Visualization, Writing – original draft, Writing – review & editing. JP: Investigation, Project administration, Visualization, Writing – original draft, Writing – review & editing. AZ: Writing – review & editing. OB: Writing – review & editing. KK: Writing – review & editing. TJ: Writing – review & editing. GA: Conceptualization, Methodology, Resources, Supervision, Writing – review & editing.
